# Supplementary material for: Long Non-Coding RNA CRYBG3 Promotes Lung Cancer Metastasis via Activating the eEF1A1/MDM2/MTBP Axis
Source: Int J Mol Sci. 2021 Mar 22;22(6):3211. doi: 10.3390/ijms22063211 (PMC8048704; doi:10.3390/ijms22063211)
Supplement: Supplementary file 1 [file ijms-22-03211-s001.pdf]

International Journal of
Molecular Sciences

MDPI

Supplementary Materials

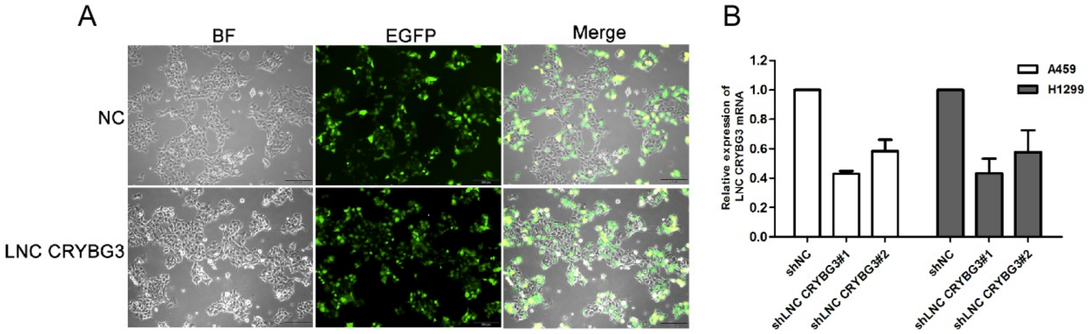

Figure S1. (A) The fluorescence images of HCC827 cells transfected with LNC CRYBG3 adenovirus particles with EGFP reporter for 24h. (**B**) A549 and H1299 cells were transfected with two kinds of LNC CRYBG3 shRNA lentivirus particles for 24 h and then screened with medium containing 2 g/mL of puromycin and double-checked with RT-PCR. The result showed that shLNC CRYBG3#1 interference effect was better than shLNC CRYBG3#2. So shLNC CRYBG3#1 was selected in the following experiments.

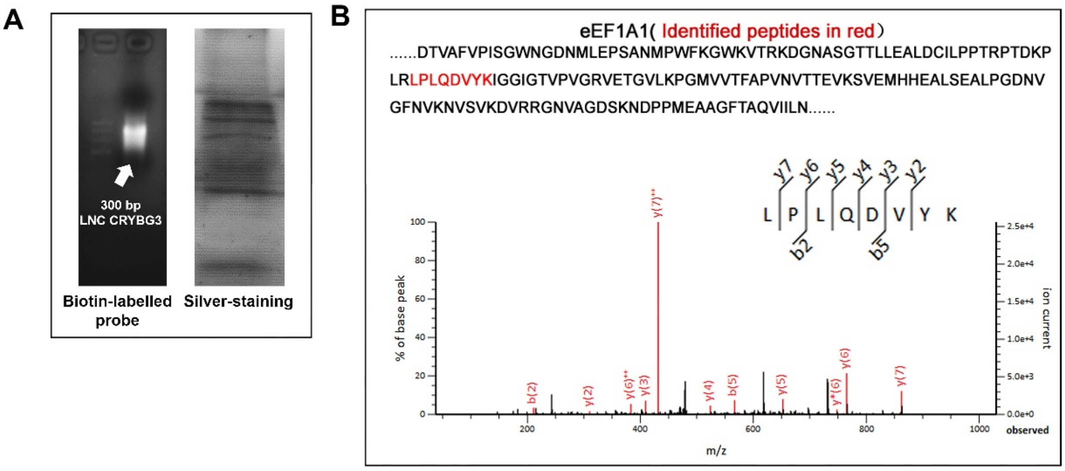

Figure S2. (A), Agarose gel electrophoresis imaging of biotin-labeled LNC CRYBG3 and SDS-PAGE silver staining of LNC CRYBG3 complexes captured on beads isolated from RNA biotin pull-down assay. (**B**), Identification of eEF1A1 using mass spectrometry analysis.

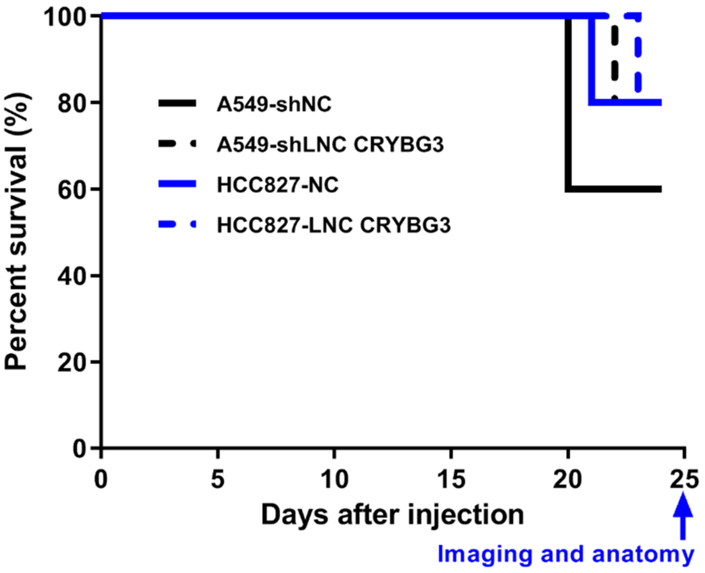

Figure S3. The survival curves of the mice in each group (n = 5). All mice were imaged and dissected at the 25th day after injection.

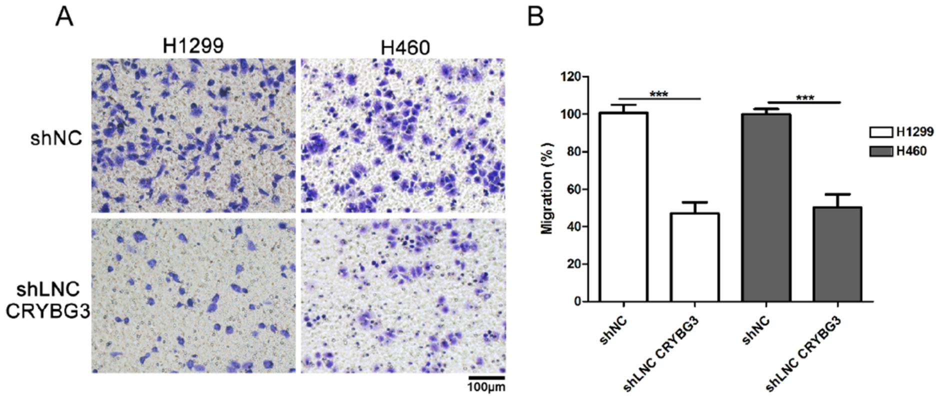

Figure S4. (**A-B**), Transwell migration analysis of H1299 (p53 mutant) and H460 (p53 deficient) cells transfected with LNC CRYBG3 shRNA (shLNC CRYBG3). The result revealed that the role of LNC CRYBG3 in regulating metastasis is not related to p53 status. Data are represented as means ± SD (***p < 0.001).

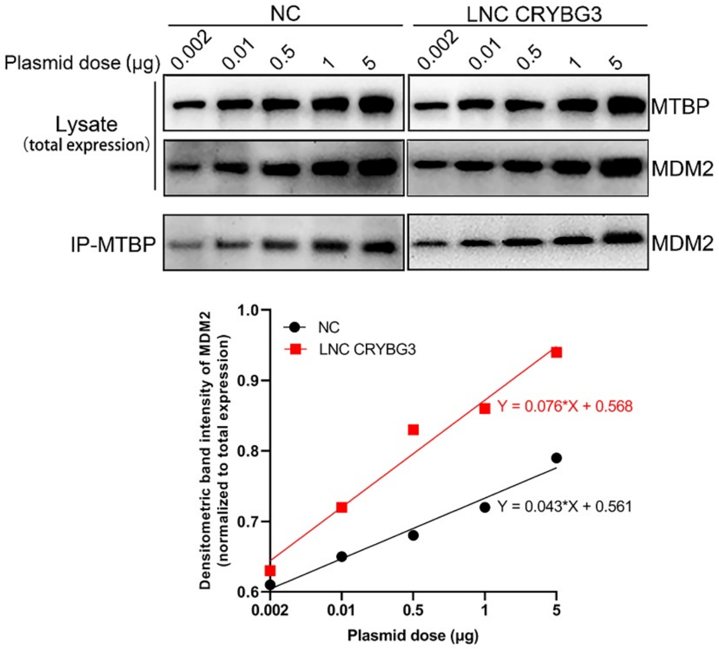

Figure S5. HCC827-NC and HCC827-LNC CRYBG3 cells were co-transfected with MDM2 and MTBP plasmids (with plasmid dose of 0.002μg, 0.01μg, 0.5μg, 1μg and 5μg) for 24h, then co-IP assay was performed using MTBP antibody-conjugated microbeads to detect MDM2 and MTBP interaction for each transfection case. The immunoblot band intensity of the MTBP combined to MDM2 was normalized to total MDM2 in lysate (measured by image J), then the interaction strength vs total expression curve was analyzed by GraphPad Prism 8.3.0.
